# Human Cytomegalovirus and Epstein–Barr Virus Infection in Inflammatory Bowel Disease: Crossing the Diagnostic Barrier for Appropriate Management

**DOI:** 10.3390/biomedicines13122915

**Published:** 2025-11-28

**Authors:** Rachele Ciccocioppo, Federico Caldart, Antonio Piralla, Elena Betti, Luca Frulloni, Antonio Di Sabatino, Fausto Baldanti

**Affiliations:** 1 Gastroenterology & Endoscopy Unit, Department of Medicine and Science of Ageing, Ospedale Clinicizzato SS. Annunziata Chieti, University Gabriele d’Annunzio of Chieti, via dei Vestini, 66100 Chieti, Italy; 2 Gastroenterology Unit, Department of Medicine, Verona University Hospital, Piazzale L.A. Scuro 9, 37134 Verona, Italy; 3 Microbiology and Virology Department, Fondazione I.R.C.C.S. Policlinico San Matteo, Piazzale Golgi, 19, 27100 Pavia, Italy; 4 Internal Medicine Unit, Department of Internal Medicine and Therapeutics, Fondazione I.R.C.C.S. Policlinico San Matteo, University of Pavia, Piazzale Golgi, 19, 27100 Pavia, Italy; 5 Department of Clinical, Surgical, Diagnostic and Paediatric Sciences, University of Pavia, Piazzale Golgi, 19, 27100 Pavia, Italy

**Keywords:** human cytomegalovirus, Epstein–Barr virus, human herpesvirus, diagnosis, inflammatory bowel disease, management, viral load

## Abstract

Human cytomegalovirus and Epstein–Barr virus are widely distributed viruses that, upon primary infection, establish a lifelong latency in immunocompetent subjects, whereas, in immunocompromised patients, they may give rise to secondary infection. In the latter case, either virus may cause systemic or end-organ disease that, if localized into the intestinal tract, is almost impossible to distinguish from a flare of inflammatory bowel disease. Optimal management of this condition begins with using the right diagnostic test and definitions. Viral load quantification on biological samples (blood or tissue) by real time-polymerase chain reaction not only shows the best diagnostic accuracy but also makes it possible to distinguish between viral reactivation, infection, and disease, the clinical approach to which differs substantially. A crucial role is also played by the host virus-specific T-cell immunity, monitoring of which may improve patient management. In this regard, the advent of new therapeutic and vaccination tools, as in the transplantation field, is expected to improve patients’ outcome.

## 1. Introduction

The gut mucosa is endowed with rapid epithelial turnover and intense immune cell traffic that contribute to the maintenance of the gut barrier integrity and guarantee a remarkable capacity for repair when tissue damage occurs [1]. However, there are conditions where the healing process is impaired, thus resulting in lifelong debilitating conditions, such as inflammatory bowel disease (IBD), namely Crohn’s disease (CD) and ulcerative colitis (UC) [2]. Despite the advent of new therapeutic tools, a high percentage of patients do not respond to or lose the response to ongoing therapy [3]. A proportion (the exact proportion of which is not well-established) of refractoriness to ongoing therapy is caused by opportunistic viral infections, including those caused by human cytomegalovirus (hCMV) and Epstein–Barr virus (EBV). Both viruses are ubiquitous worldwide, infecting approximately 90% of the human population [4,5], and similar rates were found in the IBD population [6,7]. Importantly, they can establish latency in target cells and reactivate in cases of reduced host immunity [8], giving rise to both systemic and/or end-organ disease [9]. Localization in the gut mucosa represents a clinical challenge in the IBD setting, since it makes it difficult to distinguish disease relapse from superimposed viral colitis. In addition, uncertainty on the technique to be applied to achieve a correct diagnosis [10], together with poor knowledge about the interaction between the virus and the host, make the management of this condition very challenging. As a result, both hCMV and EBV infections worsen the prognosis of IBD and are associated with an increased rate of morbidity, hospitalization, and surgery [11,12]. Further reasons contributing to jeopardizing the knowledge on hCMV and EBV infection in IBD are the lack of clear definitions and standardization of virological monitoring, thus leading to misdiagnosis and underestimation of this condition. In accordance with the ECCO guidelines on infections in IBD [13], the evidence regarding the diagnosis and treatment of EBV and hCMV is very limited. Drawing on our personal experience and interdisciplinary collaboration, we aimed to outline an appropriate diagnostic pathway for defining and managing IBD patients and to stimulate discussion about the role of these viruses in this clinical context.

## 2. hCMV and EBV Infection

Both EBV (HHV-4, gamma-herpes virus family) and hCMV (HHV-5, beta-herpes virus family) primary infection usually occurs during early life and young adulthood [14,15]. This is often asymptomatic, with most symptomatic cases presenting with a self-limited disease, called “mononucleosis”, characterized by fever, malaise, leukopenia, low platelet count, and elevated liver enzymes and C-reactive protein [16]. Following primary infection, both viruses are endowed with the ability to establish lifelong latency in specific host cell populations [17], such as white blood cells progenitors, epithelial and endothelial cells for the hCMV [14], and resting memory B-cells for the EBV [18]. In addition, hCMV has several immune-evasion mechanisms, among which a functional homologue of human interleukin-10 gives the virus the ability to evade immune recognition and clearance [19]. Regarding EBV latency, only 10 proteins can be expressed, including two types of noncoding small RNAs (EBER1 and EBER2), six nuclear proteins (EBNA1, EBNA2, EBNA3A, EBNA3B, EBNA3C, EBNA5), and two membrane proteins (LMP1 and LMP2), which allow EBV to escape the virus-specific T-cell-mediated immune response [5,15]. It should be emphasized that EBV displays at least three different latent phase programs [20] and that each of them has been associated with the development of lymphatic or epithelial neoplasms [5,15,18], such as B- or T/NK-cell lymphomas, nasopharyngeal carcinoma, post-transplant lymphoproliferative disease (PTLD), hemophagocytic lymphohistiocytosis, and gastric adenocarcinoma. In contrast to latency, the lytic phase is characterized by the complete expression of viral proteins, including those needed for virion assembly and envelopment, and epithelial cells are usually used by both viruses to spread into the organ/body of the host [8,21]. Replication and latency are the result of a delicate balance between the viral lytic–latent phases and the host immune response [15,22]. Loss of virus-specific T-cell control and/or changes in the differentiation/activation state of cells that retain the viral genome can lead to the reactivation of latent infection and production of viral progeny. In immunocompetent subjects, reactivation may occur sporadically and is usually clinically silent. By contrast, in immunocompromised people, i.e., those suffering from primary immunodeficiencies, therapeutic immunosuppression (e.g., solid organ or hematopoietic stem cell transplants, autoimmune diseases), those with inborn errors of immunity (IEI), malnutrition, or the active phase of underlying disease, reactivation of viral infection through the switch from the latent to lytic phase leads to secondary infection. This may cause either systemic/end-organ disease localized in virtually all tissues and apparatuses [9,23] or chronic active secondary infection, characterized by prolonged fever, hepatosplenomegaly, and pancytopenia [15]. Definitions related to hCMV and EBV infections are shown in Table 1.

## 3. hCMV and EBV Infection in IBD

The rate and age of hCMV and EBV primary infection in IBD patients are similar to those in the general population, with early infancy and adolescence being the most frequent ages at which the infection is contracted [6,7]. EBV primary infection commonly occurs in young patients under immunosuppressive therapy especially with thiopurines. Serological screening immunoglobulin (Ig)G and IgM is mandatory before starting treatment [24]. This is due to the possibility of developing lymphoma [25].

Apart from this rare event, the most common condition in clinical practice is secondary infection. This represents a major challenge in the setting of IBD, since both viruses take advantage of local immune weakness/inflammation [26,27], thus giving rise to end-organ disease localized at the same site as the primary disease. It is well-recognized that hCMV and EBV secondary infection causes exacerbation of symptoms, lack of response to therapy, and unfavorable clinical outcomes [11,12]. Nevertheless, the detection rate of these infections in IBD patients varies considerably [28,29,30,31,32]. The principal factors contributing to this discrepancy are the retrospective design of most studies and the heterogeneity of patient cohorts and of diagnostic techniques employed that yield qualitative data on the expression of specific viral proteins rather than a quantitative assessment of the viral load. Furthermore, the majority of studies focus on hCMV infection, with scant or no attention paid to the possibility of simultaneous reactivation of both viruses or EBV alone. Looking at the European Crohn’s and Colitis Organization (ECCO) guidelines [13], positivity for viral antigens/copies on immunohistochemistry (IHC) or molecular assay, respectively, together with signs and/or symptoms of active disease are considered sufficient to confirm active viral colitis. In our opinion, this framework is the main cause of misdiagnosis/underdiagnosis of hCMV and EBV secondary infection, since it does not enable differentiation between latency and reactivation and between infection and disease.

## 4. Diagnosis of EBV and hCMV Infection

**Figure 1:**
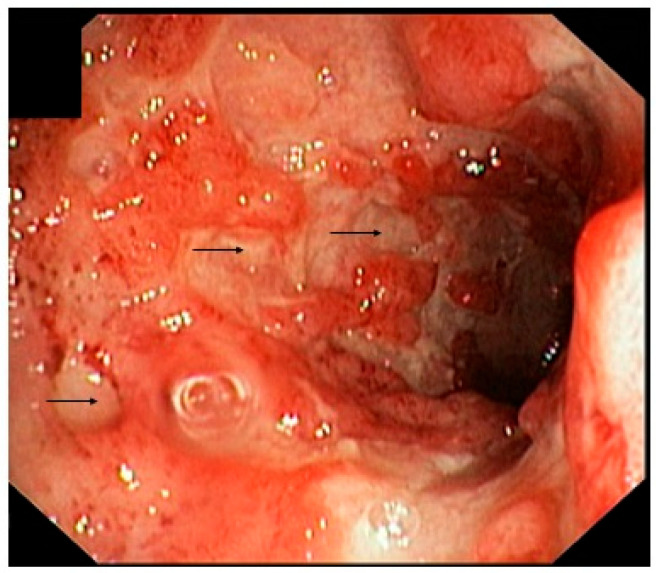
**Endoscopic features of superimposed viral colitis in a case of ulcerative colitis refractory to ongoing therapy.** Presence of severe lesions characterized by deep ulcerations with exposure of the underlying muscular layer (black arrows), surrounded by nodular-cobblestone and spontaneously bleeding zones, embedded in edematous and erythematous mucosa, are clearly evident in a representative case of superimposed human cytomegalovirus and Epstein–Barr virus colitis that are indistinguishable from those of a flare of the underlying disease.

The techniques used to detect hCMV and EBV infection may be direct, i.e., the possibility of finding the proteins and/or nucleic acid in peripheral blood and/or a specific body fluid, tissue, or organ, or indirect, i.e., measuring specific antivirus antibodies in the serum [33,34]. In this case, an enzyme­linked immunosorbent assay (ELISA) is usually performed on a serum sample to search for both IgM and IgG classes. The presence of specific antibodies belonging to the IgG class identifies prior exposure to the virus, although it is of little use for diagnosing secondary infection or disease. IgM class antibodies can be detected in primary infection and usually take a few weeks to one year to disappear; notably, they are often absent or delayed during reactivation, especially in immunocompromised patients [35]. Therefore, serology, also according with the ECCO guidelines [13], is recommended as a screening test in young people before starting immunosuppressive therapy, especially with thiopurines. Outside of this context, the most frequent scenario is the need to differentiate between a flare of the underlying disease and viral secondary infection/disease. Endoscopic findings are useless, since macroscopic lesions, such as erythema, reduced vascular pattern, friability, erosions, ulcers, bleeding, and pseudopolyps are characteristic of either IBD activity or superimposed viral colitis [36,37]. However, it is conceivable that the ability of these viruses to destroy epithelial cells due to their active replication may lead to deep ulcerations (punched-out ulcers) with exposure of underlying muscolaris mucosa (Figure 1). In any case, the severity of the endoscopic features and/or the refractoriness to ongoing treatment should prompt the suspicion of a superimposed viral infection/disease [38]. Following common clinical practice, endoscopic specimens are taken and directly fixed in formalin for histological examination. Table 2 provides a list of the direct techniques available for detecting hCMV and EBV infection in tissue samples and body fluids with their advantages and disadvantages.

### 4.1. Cell Culture and Antigenemia

Both tests are obsolete and are reserved for the detection of hCMV infection [39]. Briefly, cell culture may be performed only in tertiary laboratories and is usually applied to assess viral strains and their resistance to antiviral drugs. However, the hCMV-induced cytopathic effect on fibroblast cell culture takes up to three weeks to manifest; therefore, this method is of scarce utility in clinical practice. A useful alternative is rapid cell culture using monoclonal antibodies against viral immediate-early antigens, the results of which are available within one- to two days. Testing for hCMV pp65 antigenemia by immunofluorescence or immunochemistry on circulating polymorphonuclear neutrophils shows a sensitivity of 47% and specificity of 82% and allows a semi-quantitative measure of viral load [40]. Both techniques are demanding and do not reflect the viral load in the gut mucosa; so, they have been replaced by molecular assays.

### 4.2. Traditional Histology, Immunohistochemistry, and in Situ Hybridization

The only histologic feature suggestive of viral infection is the presence of intranuclear “owl’s eye” inclusions, visible on routine hematoxylin and eosin staining and consisting of basophilic intranuclear inclusion bodies surrounded by a clear halo that are characteristic of hCMV infection [41]. Cytomegalic inclusion bodies are rarely found, and hematoxylin and eosin staining followed by IHC on colonic biopsies have a low sensitivity [42,43], as also reported in a recent metanalysis (overall pooled sensitivity 12.5% [95% CI: 3.6–21.4], 34.6% if compared to IHC [95% CI: 13.8–55.4], 4.7% if compared to tissue PCR [95% CI: 1.2–17.1]) [44]. To improve the IHC diagnostic accuracy, it has been proposed that a cutoff of at least five hCMV+ cells/higher power field [45] should be used, whereas patients with two or more hCMV+ cells have been found to display a higher risk of colectomy [46]. However, Kredel et al. stated that only one positive cell is sufficient, despite having a high pre-test probability since they performed IHC in UC patients with high clinical suspicion of hCMV colitis [47]. Furthermore, the best area for collecting colonic tissue specimens and the quantity needed to identify viral infection have not yet been fully validated. A model has been proposed to standardize mucosal sampling (at least 11 biopsies in UC and 16 biopsies in CD) to achieve 80% probability of hCMV detection [48]. Remarkably, no data on the diagnostic accuracy of traditional histology for EBV detection are available, whereas IHC, using monoclonal antibodies to specific viral antigens, such as LPM1-1, seems to increase the diagnostic sensitivity and specificity by about 30% [49]. On formalin-fixed paraffin-embedded specimens, it is also possible to perform in situ hybridization (ISH) using specific probes for EBV constituents, such as EBER and EBNA, that are usually found in CD20^+^ B-cells and appears to have high sensitivity [50]. Although this technique is widely regarded as the gold standard, it should be borne in mind that histological sections are only 3–4 µm in thickness, whereas the diameter of a small intestinal villus is about 138 ± 15 µm, and that of a colonic crypt is about 50 ± 5 µm, thus highlighting the possible underestimation of the viral positivity, similarly to that found when evaluating gut mucosal epithelial turnover [51]. In line with this finding, our previous evidence of the presence of viral DNA in almost all epithelial cells—other than intraepithelial lymphocytes and lamina propria mononuclear cells—isolated from colonic mucosa of IBD patients refractory to ongoing therapy as assessed by real-time quantitative polymerase chain reaction (qPCR), whilst only rare positivity was evident at IHC/ISH examination in immune cells, and never in epithelial cells, despite the fact that the samples were harvested from the same area [52], strengthens this concept. Further explanations for this discrepancy may lie in the possibility that the antigen/s tested was/were not expressed in the phase of the viral life cycle occurring when the biopsies were taken and/or that the epithelial cell burst caused by viral replication does not allow them to be stained. Finally, the identification of a positive cell by IHC/ISH does not provide any information on the possible pathogenic role of the virus, since it may occur even in control subjects following periodic reactivation of the virus to test the host’s immune competence.

### 4.3. Molecular Assays

Real-time qPCR is currently the “gold standard” method for detecting and quantifying both hCMV and EBV DNA load in almost all clinical specimens, including gut mucosa [53]. This is a highly specific technique that enables the quantitative measurement of viral load, which is essential for accurately determining the role of the virus in a given clinical context. Indeed, consensus thresholds for hCMV load have been proposed for initiating pre-emptive antiviral treatment in solid organ as well as in hematopoietic stem cell transplanted patients [35,54]. With regard to IBD, only a limited number of studies employed this technique usually focusing only on hCMV infection, with different cut-off values for mucosal or blood viral load being put forth to distinguish between latent and active infection, as well as to ascertain the necessity for antiviral therapy [55,56,57]. Our group has previously highlighted the discrepancy between the positivity for both hCMV and EBV infections found by real-time qPCR and that found by using IHC or ISH, with a sensitivity of 30% and specificity of 80% for the latter techniques, despite the fact that the mucosal biopsy site was the same [58]. Importantly, the assessment of viral load by real-time qPCR on freshly collected mucosal samples allowed us also to establish a threshold value for distinguishing amongst end-organ disease, secondary infection, and reactivation. More in depth, a peak value ≥ 10^3^ DNA copies/10^5^ cells invariably indicated the presence of a superimposed viral colitis as end-organ disease, whereas a value of 10^2−3^ DNA copies/10^5^ cells suggested secondary infection, and a value < 10^2^ DNA copies/10^5^ cells might be related to the occasional reactivation that occurs also in healthy people [58]. To reinforce the concept that the occurrence of colonic disease/secondary infection/reactivation is the most frequent localization occurring in the IBD setting, we demonstrated that viral copies were only rarely found in peripheral blood and usually when the mucosal value was ≥10^5^ copies/10^5^ cells [58]. In our opinion, therefore, the presence of detectable viral copies in the peripheral blood, independently from the number, is indicative of systemic disease and it does not change the clinical approach. In another, albeit retrospective study on 229 subjects, it was suggested that a blood DNA cut-off value of >250 DNA copies/mL can be used, although it was confirmed to have low sensitivity for diagnosing hCMV colitis [40]. This finding is of meaningful clinical value, since neither IHC/ISH on mucosal samples nor real-time qPCR on peripheral blood samples have any diagnostic value for differentiating a flare of the underlying disease from a superimposed viral colitis. In contrast, real-time qPCR carried out on mucosal samples is highly sensitive (threshold of one viral copy) and can be obtained within 24 h, a timeframe that allows for rapid resolution of important clinical issues. An added value is the possibility of performing serial determination to obtain information on the kinetics of the viral replication over time, although its interval needs to be defined through ad hoc studies. In case of unavailability of prompt/local analysis on freshly harvested specimens, their cryopreservation and shipment at −80 °C is strongly recommended. It is worth noting that, following the first worldwide collaborative study carried out to establish an International Standard for hCMV and EBV nucleic acid amplification techniques, an assigned concentration of ~5 × 10^6^ International Units per mL was identified [59]. The introduction of this value facilitated the harmonization of results, thereby establishing a uniform reporting system and a traceable measurement framework that is independent of the method used.

Finally, PCR assay on fresh stool samples may prove to be an easy, non-invasive, and convenient tool [60], as also recommended by the ECCO [13]. However, it is a qualitative test, not useful for distinguishing between colonic reactivation, infection, and colitis, and it has only been applied to detect hCMV DNA.

### 4.4. Genotyping

At variance with the data on the distribution of pathogenic hCMV strains in patients with congenital infection, solid organ or hematopoietic stem cell transplantation, and those with acquired immunodeficiency syndrome [61], only limited data are available on hCMV genotyping in UC and none on EBV. In a study by Nahar and coworkers, glycoproteins B1, N3, and H2 were the most frequent genotypes found in UC, and a correlation was reported between the gB1 and gH2 genes and symptom severity [62]. Therefore, a genotypic analysis in a large IBD population is warranted to elucidate the role, if any, of different hCMV strains and their change over time.

### 4.5. Immunological Assays

The ELISpot assay measures the frequency of CD4^+^ and CD8^+^ T cells producing interferon-γ in response to virus-specific peptides [63]. In a previous study, we showed that impaired EBV-, but not hCMV-, specific T-cell immunity was evident in UC patients, mostly in those refractory to therapy [64]. Nowadays, several commercial kits and automated reader machines are available, whose application has become crucial for optimizing preventive and pre-emptive therapy in the setting of transplant recipients [65]. Flow cytometry can also be used to run an hCMV T-cell immunity panel by using intracellular cytokine staining. The advantage of this assay is that a range of cytokines and cell surface molecules may be applied to provide quantitative and qualitative measurements of hCMV-specific T-cells.

## 5. Treatment of hCMV and EBV Infection

Unfortunately, both the diagnosis and treatment of hCMV and especially of EBV infection in the context of IBD are controversial, and several clinical and viral variables should be considered to establish optimal patient management.

### 5.1. Therapeutic Options for HCMV Infection

**Figure 2:**
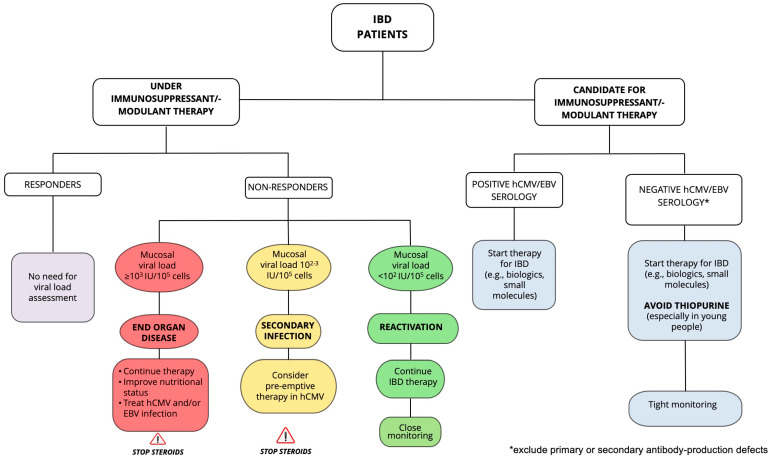
**Proposed algorithm for the management of human cytomegalovirus and Epstein–Barr virus infections in inflammatory bowel disease**. First, IBD patients are grouped according to immune-suppressant/-modulant therapy: whether ongoing or candidate for. For those who are under treatment, a mucosal viral load assessment is required only in case of no response. In this case, prompt antiviral treatment (see the text) is indicated for those with a mucosal viral load exceeding 10^3^ copies/10^5^ cells (or equivalent), since it means the presence of a superimposed viral colitis (end-organ disease), independently from the presence or absence of detectable viral copies in the peripheral blood. For those with a mucosal viral load within 10^2−3^ copies/10^5^ cells (or equivalent), we strongly suggest starting pre-emptive therapy in the case of hCMV infection, since it means the presence of secondary infection that might rapidly evolve to end-organ disease, whilst no therapeutic options are available for EBV infection. However, in either case of end-organ disease and secondary infection, quick tapering and then discontinuation of steroids are strictly recommended, while supporting the nutritional status. No modification of the treatment is needed for those with a mucosal viral load lower than 10^2^ copies/10^5^ cells (or equivalent), even though a close monitoring is required aimed at determining the possible shift of viral reactivation to secondary infection/viral colitis and consequent timely initiation of an antiviral treatment. For those who are candidates for immune-suppressant/-modulant treatment, serological screening for viral infections is required and, in the case of negative EBV serology, thiopurines must be avoided, and a strict monitoring with periodic assessment of the peripheral blood viral load and imaging is required. *Abbreviations: EBV: Epstein–Barr virus; hCMV: human cytomegalovirus; IBD: inflammatory bowel disease; IU: International Units*.

Evidence on hCMV infection in IBD patients are stronger than those on EBV, and according to the ECCO guidelines [13], the treatment consists of intravenous ganciclovir (5 mg/kg twice daily for 5- to 10 days), followed by valganciclovir (900 mg daily), until completion of a two-to-three-week course. However, we strongly recommend differentiating treatment according to the mucosal viral load, which allows distinguishing between viral reactivation, secondary infection, and end-organ disease (Figure 2). More specifically, in the case of superimposed hCMV colitis, i.e., with a mucosal viral load ≥ 10^3^ copies/10^5^ cells (or equivalent), regardless of the presence or absence of detectable viral DNA in the peripheral blood, starting with antiviral agents (e.g., intravenous ganciclovir 5 mg/kg twice daily for two weeks, followed by valganciclovir 900 mg daily for further two weeks, as for immunocompromised patients) and rapid tapering and discontinuation of corticosteroids are strongly recommended. By contrast, discontinuation of monoclonal antibodies/thiopurine maybe not useful, due to their long-lasting effects, while no evidence is yet available on small molecules. Failure to achieve clinical improvement after 14 days is referred to as “resistant/refractory hCMV”, and a therapeutic switch to foscarnet or cidofovir is usually applied [66]. The recent introduction of maribavir for treatment of refractory hCMV [67], together with the possibility to use letermovir for prophylaxis in hematopoietic stem cell transplants [68] has radically changed the therapeutic landscape of hCMV infection in the setting of bone marrow and solid organ transplantation, offering patients the potential for effective anti-hCMV treatment without the high rate of adverse events often associated with traditional therapies. Translating this evidence to the field of IBD, we might state that if the tissue viral load is below the threshold of 10^3^ copies/10^5^ cells (or equivalent), close monitoring and possibly prophylactic/pre-emptive therapy, avoiding systemic steroids, should be considered. Of course, the use of immunity tests holds the potential to improve patient management thanks to better risk stratification, determination of the duration of antiviral prophylaxis, and estimation of the risk of recurrence [69,70], since hCMV infection, as well as EBV, may even resolve spontaneously thanks to the resilience of the host immunocompetence, although this event is unpredictable. Finally, if the viral load is <10^2^ DNA copies/10^5^ cells (or equivalent), no specific treatment is indicated, but monitoring is highly recommended [69].

### 5.2. Therapeutic Options for EBV Infection

If symptomatic primary EBV infection occurs in IBD patients who are not taking immunosuppressive therapy, the treatment is based on general supportive care as usual [15]. If an EBV-negative patient needs to undergo immunosuppressive therapy, we suggest adopting the recommendations for bone marrow transplantation patients, with virological monitoring by assessing the viral load in peripheral blood rather than serology, since these patients may fail to mount an antibody response. In the case of the appearance of EBV copies and especially in the case of imaging consistent with PTLD, prompt therapy with anti-CD20 monoclonal antibody (rituximab, 375 mg/m^2^ body surface intravenously every week for four weeks) should be established, while immunosuppressive therapy should be quickly reduced/discontinued [71]. Aside from this rare situation, in everyday clinical practice, we are usually faced with secondary infection. Unfortunately, even if a correct diagnosis is established, no approved medications are available for treating EBV disease, although there are several strategies to inhibit its replication, such as (i) improving the nutritional status while reducing/discontinuing immunosuppressants/steroids to restore virus-specific T-cell response; (ii) use of nucleoside analogs (acyclovir, valacyclovir, ganciclovir, valganciclovir) to inhibit viral DNA-polymerase; (iii) use of histone deacetylase inhibitors (valproic acid, nanatinostat) that trigger apoptotic cell death; (iv) use of autologous or third party EBV-specific cytotoxic T-cell therapy; and (v) monoclonal antibody (rituximab) capable of destroying cells bearing the CD20 antigen, which host the virus [72]. In our experience, rituximab was successful in rescuing patients from EBV-related end-organ disease and decreasing the mucosal viral load, albeit not in avoiding subsequent colectomy [58]. Furthermore, adoptive T-cell therapy with EBV-specific cytotoxic T lymphocytes can be safely and effectively applied [73], as also demonstrated by our group in two patients with primary immunodeficiency suffering from EBV enteritis [74]. Finally, in the case of a mucosal viral load below the threshold of 10^3^ copies/10^5^ cells (or equivalent), we suggest close monitoring while improving the nutritional status and discontinuing systemic steroids that are the main trigger of viral lytic phase [64], whereas if the viral load is <10^2^ DNA copies/10^5^ cells (or equivalent), no specific treatment is indicated, but monitoring is highly recommended (Figure 2).

### 5.3. Therapeutic Options for EBV and hCMV Simultaneous Infection

High DNA loads of EBV and hCMV have been observed in the same enterocytes, suggesting a simultaneous infection [52]. Since there is no established gold-standard therapy for either virus, current evidence suggests treating CMV first. As mentioned above, therapies targeting viral DNA polymerase (e.g., nucleoside analogs) may be helpful in managing simultaneous EBV and CMV infections. In addition, tapering steroids and improving the patient’s general and nutritional status can indirectly support the host immune response, promoting the return to a latent viral phase.

## 6. Conclusions

Both hCMV and EBV primary infection may occur in IBD patients with the same prevalence as in the general population. Serological screening, particularly for EBV, is recommended before starting immunosuppressive therapy to prevent possible life-threatening complications.

EBV and hCMV secondary infection can lead to “end-organ disease” and/or to “systemic diseases”, involving dissemination throughout peripheral blood. In IBD patients, the most common manifestation is superimposed viral colitis. In this condition, clinical, endoscopic and clinical features are indistinguishable from those of a flare of the underlying disease, making diagnosis challenging.

The only reliable diagnostic test is real-time qPCR performed on fresh or cryopreserved mucosal specimens, which allows differentiation between viral reactivation, secondary infection, and end-organ disease. In addition, assessing virus-specific T-cell immunity can help identify patients at higher risk for secondary infection and, possibly, start prophylaxis. Recent advances in therapeutics and vaccination strategies are changing the clinical management of these infections [75]. If standard IBD therapy fails, and an opportunistic infection is suspected, referral to a reference center with access to advanced diagnostics (quantitative PCR, immune evaluation) and multidisciplinary expertise is indicated. In conclusion, a structured approach combining early screening, precise diagnosis, risk stratification, and expert multidisciplinary management is essential to overcome the diagnostic challenges and improve the clinical outcomes for IBD patients with hCMV or EBV infection.

## Figures and Tables

**Table 1:** Definitions related to human cytomegalovirus and Epstein–Barr virus infections.

DEFINITION	MEANING	CLINICAL FEATURES
**Infection**	Isolation of the virus or detection of viral proteins/nucleic acid in any biological sample.	
**Primary infection**	Appearance of de novo specific antibodies in a seronegative patient, provided that passive transfer via immunoglobulin or blood products can be excluded. Detection of viral components in an individual previously found to be seronegative.	Asymptomatic in most cases. Those few symptomatic cases presenting with a self-limited disease (“mononucleosis”) commonly characterized by fever, malaise, leukopenia, low platelet count, and elevated liver enzymes and C-reactive protein.
**Secondary Infection**	Switch from the latent to lytic phase. Presence of viral DNA in a biological sample in an individual known to be seropositive.	Asymptomatic in immunocompetent hosts. Symptomatic in immunocompromised hosts.
**Re-infection**	Detection of a viral strain that is different from the strain that was the cause of the patient’s original infection.	Usually symptomatic.
**Reactivation**	Switch from the latent to lytic phase. Reactivation is assumed if the two viral strains found in two episodes of secondary infection are found to be indistinguishable.	In immunocompetent subjects, reactivation may occur sporadically and is usually clinically silent. In immunocompromised people, reactivation of viral infection may lead to end-organ or systemic disease.
**Lytic phase**	Complete expression of viral proteins, including those needed for virion assembly and envelopment.	Symptomatic (similar to primary infection).
**Latent phase**	The condition in which the virus genome is retained within the human genome in specific cell populations and viral gene expression is deeply limited, in the absence of replication of the virus.	Asymptomatic.
**Chronic active secondary infection**	Chronically active lytic phase with persistent presence of viral DNA in biological samples.	Presence of prolonged fever, malaise, hepatosplenomegaly, and pancytopenia.
**Systemic disease**	Presence of high viral load in peripheral blood.	Presence of systemic involvement including fever, malaise, fatigue, and eventually splenomegaly, cytopenia, and lymphadenopathy.
**End-organ disease**	Presence of high viral load in biological samples from any body organ/tissue/fluid.	Presence of signs and symptoms related to the damage of the target organ.

**Table 2:** Methods for human cytomegalovirus and Epstein–Barr virus detection or specific immune response evaluation.

TECHNIQUE	TYPE OF SPECIMEN	ADVANTAGES	DISADVANTAGES
**Viral culture**	Tissue or blood	High specificity Detecting replicating virus Assessing viral strains Testing drug resistance	Up to three weeks for the results Low sensitivity Mostly in a tertiary center
**Antigenemia (pp65)**	Blood	High specificity Rapid and giving a semiquantitative measure Easy to perform	Low sensitivity False negative in case of neutropenia Not always correlated to viral load Only for hCMV
**Antibodies IgM and IgG**	Serum	Easy to perform Useful as screening before starting immunosuppressive therapy IgG useful for assessing with IgG prior exposure IgM can be detected in primary infection	IgG—no utility for the diagnosis of secondary infection or disease IgM can take a few weeks to one year to disappear May not be diagnostic in case of primary or secondary antibody production disorders
**Histopathology**	Tissue	Easy to perform Possibility of finding characteristic “owl’s eye” Immunohistochemistry and in situ hybridization improve sensitivity	Low sensitivity Time-consuming Expensive
**Real-time quantitative polymerase chain reaction** **(qRT-PCR)**	Tissue or blood	High sensitivity and specificity Quantitative measure of viral load Helpful in distinguishing end-organ/systemic disease, secondary infection, and reactivation Rapid results	Different viral load in blood vs. plasma
**Genotyping**	Tissue or blood	Potentially useful	Lack of data in IBD
**ELISpot Assay** **(immune response)**	Blood	High sensitivity Can distinguish CD4^+^ and CD8^+^ T-cell response	Expensive
**Flow cytometry** **(immune response)**	Blood	Can provide qualitative and quantitative measures Can distinguish CD4^+^ and CD8^+^ T-cell response	High-level expertise Expensive
**MHC-multimer-based assay** **(immune response)**	Blood	Helpful in determining CD8^+^ response	High-level expertise Needs cell-sorting facilities

EBV: Epstein–Barr virus; hCMV: human cytomegalovirus; IBD: inflammatory bowel disease; MHC: major histocompatibility.

## Data Availability

No new data were created or analyzed in this study.

## Abbreviations

The following abbreviations are used in this manuscript:

CD	Crohn’s disease
EBV	Epstein–Barr virus
ELISA	enzyme­linked immunosorbent assay
hCMV	human cytomegalovirus
IBD	inflammatory bowel disease
IG	immunoglobulin
IHC	immunohistochemistry
ISH	in situ hybridization
PTLD	post-transplant lymphoproliferative disease
qPCR	quantitative polymerase chain reaction
UC	ulcerative colitis
